# The Mechanism of Methylated Seed Oil on Enhancing Biological Efficacy of Topramezone on Weeds

**DOI:** 10.1371/journal.pone.0074280

**Published:** 2013-09-24

**Authors:** Jinwei Zhang, Ortrud Jaeck, Alexander Menegat, Zongjian Zhang, Roland Gerhards, Hanwen Ni

**Affiliations:** 1 College of Agriculture and Biotechnology, Weed Management laboratory, China Agricultural University, Beijing, China; 2 Institute of Plant Protection, Tianjin Academy of Agricultural Sciences, Tianjin, China; 3 Institute of Phytomedicine, Department of Weed Science, University of Hohenheim, Stuttgart, Germany; 4 The Office of National Pesticide R&D South Center R&D Center of Functional Additives, Central Research Institute of China Chemical Science and Technology, Beijing, China; United States Department of Agriculture, United States of America

## Abstract

Methylated seed oil (MSO) is a recommended adjuvant for the newly registered herbicide topramezone in China and also in other countries of the world, but the mechanism of MSO enhancing topramezone efficacy is still not clear. Greenhouse and laboratory experiments were conducted to determine the effects of MSO on efficacy, solution property, droplet spread and evaporation, active ingredient deposition, foliar absorption and translocation of topramezone applied to giant foxtail (*Setaria faberi* Herrm.) and velvetleaf (*Abutilon theophrasti* Medic.). Experimental results showed that 0.3% MSO enhanced the efficacy of topramezone by 1.5-fold on giant foxtail and by 1.0-fold on velvetleaf. When this herbicide was mixed with MSO, its solution surface tension and leaf contact angle decreased significantly, its spread areas on weed leaf surfaces increased significantly, its wetting time was shortened on giant foxtail but not changed on velvetleaf, and less of its active ingredient crystal was observed on the treated weed leaf surfaces. MSO increased the absorption of topramezone by 68.9% for giant foxtail and by 45.9% for velvetleaf 24 hours after treatment. It also apparently promoted the translocation of this herbicide in these two weeds.

## Introduction

Almost all herbicide formulations contain adjuvants which act as important tools to improve physical aspects of herbicide application and/or to enhance biological efficacy [1], [2], [3], [4]. Research on adjuvant technology for agrochemicals has made good progress in recent years in part due to increased efforts by agrochemical manufacturers to ensure that the best adjuvants are used with their products for maximum performances. The efficacy of herbicide formulation can be expressed as a function of deposition, retention, absorption, translocation and phytotoxicity. Although adjuvants are not able to directly affect inherent herbicide toxicity, they can significantly alter each of the preceding terms [4]. There are various types of adjuvants with varying degrees of effectiveness on enhancing herbicide efficacy.

Methylated seed oil (MSO) is a kind of fatty acid from seed oil esterified with methyl alcohol [5]. For oil-based adjuvant, droplet spread on leaf surfaces and herbicide penetration seem to be the two predominant factors regarding the mechanism of the enhancement in herbicide efficacy [6]. Xu et al. reported that MSO could decrease the surface tension and contact angle and then increase the wetted areas of droplets on both waxy and hairy leaves [7], [8]. Some reports have shown that MSO enhances the efficacy of several herbicides on certain weed species by increasing the absorption of the herbicides by weeds [9], [10], [11], [12], [13], [14], [15], [16]. Topramezone, a hydroxyphenylpyruvate dioxygenase inhibitor, was commercially introduced in the year of 2006 [17]. When applied as a post-emergence herbicide, it controls a wide spectrum of annual grass and broadleaf weeds [18], [19], [20], [21] and is safe to corn (*Zea mays* L.) [22], [23].

In order for the optimal weed control, topramezone should be also applied with a certain adjuvant and MSO is always recommended. This herbicide has good field performance when applied with the MSO adjuvant which is made from soybean oil in China [24], [25], [26]. Some other reports also presented that a good efficacy could be achieved when this herbicide was tank-mixed with MSO adjuvant [27], [28], [29]. However, little information has been provided about the mechanism of MSO enhancing the biological efficacy of topramezone on weeds. Only Grossmann and Ehrhardt reported that a kind of MSO adjuvant Dash HC could significantly increase the foliar absorption of topramezone by plants [17].

The objective of this study was to determine the mechanism of MSO enhancing the biological efficacy of topramezone through investigating effects of MSO on solution property, droplet behavior on weed leaf surface, active ingredient absorption by weed leaves and translocation in weed plants.

## Materials and Methods

### Materials Used and Plant Growth Conditions

Topramezone commercial formulation was 336 g L^−1^ SC provided by BASF Co., Ltd. Adjuvant MSO (GY-HMax, methylated soybean soil) was provided by the Central Research Institute of China Chemical Science and Technology, which is recommended by BASF as spray adjuvant for this herbicide in China. Unless indicated, the added amount of MSO was 0.3% herbicide solution (v/v).

A dicotyledonous weed velvetleaf (*Abutilon theophrasti* Medic.) and a monocotyledonous weed giant foxtail (*Setaria faberi* Herrm.) were used in this study. The weed seeds provided by Herbiseed Co., UK were pre-germinated in plastic pots (11×11×6 cm) filled with vermiculite (2–3 cm) in greenhouse (25/20±1°C day/night, additional light 122 µmol m^−2^ s^−1^ for 12 h, and 55±10% RH). After germination, velvetleaf seedlings were transplanted into 12-cm-diam plastic pots (3 plants per pot) and giant foxtail seedlings were transplanted into 7×7 cm paper pots (4 plants per pot). All pots were filled with the mixture of vermiculite: peat: clay 1∶1∶1 (by volume) and cultivated under the above-described condition. The plants were watered daily with tap water. No watering was done for 24 hours after herbicide application.

### Efficacy Enhancement of MSO on Topramezone

At the 3- to 4-leaf stage of weeds, topramezone was applied alone or tank-mixed with MSO at 12 series doses for giant foxtail and 10 series doses for velvetleaf. The dose ranges are given in Table 1. Plants were applied with tap water as an untreated control for each weed species. Herbicide application was done with a track sprayer (Aro, Langenthal, Switzerland), which simulated a spray volume of 200 L ha^−1^ (nozzle: 8002 EVS, Teejet® Spraying Systems Co., Wheaton, IL, USA) at 3.2 kPa. The experiment was completely randomized with 3 replicates and repeated once.

**Table 1:** Topramezone doses and its recommended adjuvant preparation for giant foxtail and velvetleaf.

Weed	Treatment	Dose range g a.i. ha^−1^	MSO* concentration %, v/v
Giant foxtail	Topramezone alone	0.394–806.4	0
	Topramezone with MSO	0.025–50.4	0.3
Velvetleaf	Topramezone	0.197–100.8	0
	Topramezone with MSO	0.197–100.8	0.3

* MSO is methylated seed oil.

Plants were harvested 3 weeks after application, dried at 80°C for 48 h and weighed. The following four-parameter Weilbull model [30] was used for dose-response curves (Equation 1):
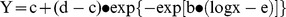
(1)where d and c denote the upper and lower limits, respectively. e is the dose where a response half-way between the upper and lower limit (ED_50_) is reached. b denotes the slope around the ED_50_ value.

To compare different models generated from dry biomass data of each treatment, the residual sum of squares of the regression analysis was assessed by an F-test for lack-of-fit. To compare the efficacy difference between the two treatments of topramezone applied alone and mixed with MSO, the relative potency (RP) (Equation 2) of two treatment curves was calculated based on their ED_90_ values according to Ritz's method [31].
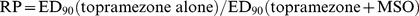
(2)


### Surface Tension and Contact Angle

Topramezone solutions were prepared at the concentration of 0.126 g a.i. L^−1^, which corresponded to 25.2 g a.i. ha^−1^ at 200 L ha^−1^. Solution Surface tensions of topramezone alone, topramezone with MSO and deionized water were measured with a contact angle/surface tensionmeter (JC2000C1, Shanghai Zhongchen Digital Technology Equipment Co., LTD, Shanghai, China).

The fourth leaves of velvetleaf and giant foxtail were removed at the 4- to 5-leaf stage. The leaves of giant foxtail were cut into about 2 cm-long segments. The segment of giant foxtail or the blade of velvetleaf was fixed onto a glass slide with double-sided adhesive tape. Adaxial surface was outward. Two 1 µL-droplets of the tested solutions were dropped onto one blade or segment with a microsyringe. Contact angles of the droplets were measured with the above mentioned instrument. Measurements of 10 droplets were carried out for each weed species.

### Spread and Evaporation of Topramezone Droplet on Leaf Surfaces

Tested leaf samples and topramezone solutions were prepared as same as in the above-mentioned surface tension and contact angle measuring experiment. In order to distinguish droplet residual borders, 0.5 mg ml^−1^ black color (Brilliant Black BN E151, produced by Ringer Kuhlmann, Hamburg, Germany) was blended into the treatment solutions. A leaf segment of giant foxtail or a whole velvetleaf blade was fixed onto a glass slide with double-sided adhesive tape. For giant foxtail, 1 droplet of topramezone alone, topramezone with MSO and deionized water was dropped onto 1 leaf segment separately with a volumetric pipette (Eppendorf, Germany). For velvetleaf, 1 droplet of the three treatments was randomly dropped onto one blade. The droplet volume was 2 µL. Time from the moment of the droplet deposited onto the leaf surface to it completely evaporated was recorded with a stopwatch and the picture of each treated leaf was taken using a camera (SAMSUNG EX1, Samung Electronics Co., DC, South Korea). This procedure was conducted under the condition of temperature at 29±1°C and 45±10% RH. The spread area was determined by tracing the marked outline of the droplet spread on the leaf surface using the free select tool of GIMP software (version 2.8). An integrated index (λ) was used to comprehensively characterize the droplet spread and evaporation and defined as the product of the spread area and the evaporation time of a droplet on a leaf surface [7], [8].

### Deposition of Topramezone on Leaf Surfaces

Topramezone solutions were prepared as same as in the above-mentioned surface tension and contact angle measuring experiment. Sample plants were cultivated in the greenhouse condition as described above. One 2 µL-droplet of the prepared solution was dropped onto the adaxial side of the fourth leaf of giant foxtail or velvetleaf with a volumetric pipette when these two weeds grow up to 4- to 5-leaf stage. About 1 cm-long leaf fragment treated with the herbicide solution was cut from the plants after the droplet completely evaporated and then was fixed onto a specimen holder of microscope with carbon double-side tape (NISSHIN EM. Co., Ltd. Tokyo, Japan). The deposition state of topramezone active ingredient on leaf surface was immediately observed under an environment scanning electron microscope (FEI Quanta 200, Czech Republic) at low vacuum mode at 12.5 kV. Pictures were taken at 1000×magnification.

### Absorption of Topramezone by Weed Leaves

Topramezone solutions were prepared similar as in the above-mentioned surface tension and contact angle measuring experiment and the concentration was 0.504 g a.i. L^−1^, which corresponded to 100.8 g a.i. ha^−1^ at 200 L ha^−1^. Three 2 µL-droplets of the prepared solutions were dropped onto the adaxial side of the flag leaf of each giant foxtail plant (at flowering stage) and the fourth leaf of velvetleaf plant (at 4- to 5-leaf stage) with a volumetric pipette. Every treatment was repeated 20 times for giant foxtail and 12 times for velvetleaf. Total leaf sample weight was around 1–2 g. Treated leaves were harvested at 2, 6, 24 and 48 hours after application (HAT). All harvested leaves were firstly washed with 70 ml deionised water for 15- to 20-s and then rinsed with 30 ml deionised water for 5- to 10-s. After being washed, the sample leaves of each treatment were immersed into 25 ml chloroform for 5-to 10-s to dissolve cuticle waxes and to remove herbicide retained on the cuticle referencing Beckett and Stoller's method [32].

After being washed by chloroform, the leaves were cut into small pieces and put into a 50-ml centrifuge tube. Acetonitrile solution of 15 ml including 2% formic acid v/v was added to the tube. The plant sample was ground with an Ultra-Turrax (IKA®-Werke GmbH & Co. KG, Staufen, Germany) for around 2 min. Sodium chloride of 1 g and anhydrous magnesium sulfate of 4 g were added to the ground sample. Then the tube was capped and immediately vortexed vigorously for 1.5 min. The sample stood for 2 h at room temperature to extract the herbicide completely from the leaf sample and the water could be absorbed completely by anhydrous magnesium sulfate. Next, the tube was centrifuged for 5 min at 4000 rpm and then 1.5 ml of the upper layer was accurately removed using a volumetric pipette and transferred to a new 2-ml centrifuge tube. Primary secondary amine of 35 mg, graphitized carbon black of 15 mg and anhydrous magnesium sulfate of 150 mg were added to this tube. The tube was then vortexed vigorously for 1.5 min and then centrifuged for 3 min at 8000 rpm. Finally the resulting aqueous (top) layer was filtered through 0.22- µm nylon syringe filters. The extracted solution of 10 µL was injected into the UPLC-MS/MS system. The UPLC-MS/MS analytical conditions were according to Li's method [33]. Peak area data of topramezone alone and mixed with adjuvant MSO treatments at different HATs were compared.

### Translocation of Topramezone in Weeds Plants

The fourth leaf of both giant foxtail and velvetleaf plants were coated with aluminum paper completely when these two weeds grew up to 4-leaf stage. Topramezone alone or mixed with MSO was applied at the rate of 25.2 g a.i. ha^−1^ on whole tested plants with the track sprayer mentioned above. When the droplets were dried on the leaf surfaces, around 10–15 min after herbicide application, the coated aluminum paper was taken off. Maximum quantum efficiency (Fv/Fm) of PS II of the fourth leaf was measured with an IMAGING-PAM M-Series Chlorophyll Fluorometer (Heinz Walz GmbH, Germany) before herbicide application and 2, 3, 4 and 5 days after treatment. Fv/Fm of PS II indicated the ratio of variable fluorescence (Fm – Fo) and maximum fluorescence (Fm), where Fo is dark fluorescence yield. Ten individual plants were measured for each treatment. For determination of Fo, plants were dark adapted for 30 min prior to the measurement. All measurements were conducted in a dark room under green illumination to avoid other photosynthetic active radiation except that emitted by the IMAGING PAM light source. After dark adaption, the fourth leaf of each plant was illuminated with a light saturation pulse of 580 µM m^−2^ s^−1^ and a wavelength of 450 nm for Fv/Fm determination. While measuring the Fv/Fm value, chlorophyll fluorescence images were taken using a CCD camera with a 680 nm filter.

To determine that the Fv/Fm value changes as a function of the natural logarithm of date after herbicide treatment, Fv/Fm was calculated according to the following equation 3 referencing the model of Rasmussen et al. [34], [35]:
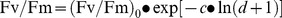
(3)where (Fv/Fm)_0_ is the estimated Fv/Fm value in untreated plant leaves before herbicide application, c is the slope parameter, d is the date after herbicide treatment.

The 95%-Confidence intervals (95%-CI) of parameter c were calculated and difference significance between different treatments was judged according to the 95%-CIs overlap with each other or not.

The surface tension and contact angle, spread and evaporation experiment were repeated once. Data of two repeated experiments were combined to analyze because there was no interaction between two experiments. Data were subjected to ANOVA and means were separated by Fisher's protected LSD test at the 5% level of probability. Absorption and translocation experiments were also conducted twice and data combined to be analyzed. All analysis and graphs were done with the statistical software R (R version 2.15.2) [36].

## Results and Discussion

### Effect of MSO on Topramezone Efficacy

**Figure 1:**
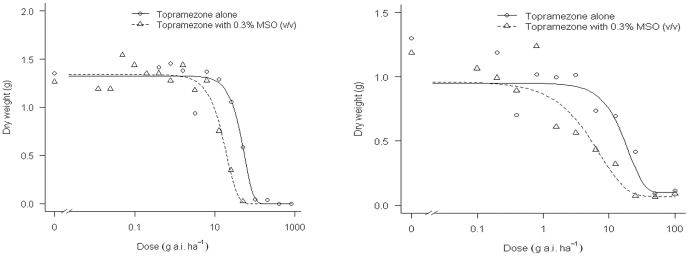
Dose-response curve of giant foxtail and velvetleaf on topramezone alone and tank-mixed with MSO. The left is the result of giant foxtail and the right is the result of velvetleaf. MSO means methylated soybean oil served as an adjuvant.

The four-parameter Weilbull model fitted well for both velvetleaf (P = 0.59) and giant foxtail (P = 0.48) curves (Fig 1). After topramezone was tank-mixed with MSO, its biological efficacy was enhanced significantly. For giant foxtail, the ED_90_ value of treatment topramezone with MSO was only 33.53 g a.i. ha^−1^, while the ED_90_ value of treatment topramezone alone was 85.06 g a.i. ha^−1^. The former treatment was 1.5-fold more effective than the latter treatment. For velvetleaf, the ED_90_ value of treatment topramezone with MSO was only 15.84 g a.i. ha^−1^, while the ED_90_ value of treatment topramezone alone was 31.56 g a.i. ha^−1^. The former treatment was 1.0-fold more effective than the latter treatment.

It should be noticed that MSO enhances herbicide efficacy on weeds and also increases phytotoxicity risk on crops. However, topramezone is highly selective between corn and weed species [17] and it has low risk on the crop when applied with MSO. Some field experimental results demonstrated that this herbicide was still highly safe to corn when it was tank-mixed with MSO [24], [25], [26].

### Effect of MSO on Surface Tension and Contact Angle of Topramezone Solution

There was no difference between surface tension of topramezone alone solution and water control treatment. After topramezone solution was mixed with MSO, its surface tension and contact angle on weed leaves decreased significantly (Table 2, 3). The surface tension decreased around 50%. This result is similar to the results of Xu et al., which demonstrated that MSO decreased the surface tension and contact angle of distilled water on the waxy leaves [7]. The decrease of the contact angle was higher on velvetleaf (26.9%) than on giant foxtail (19.0%).

**Table 2:** Effect of MSO on the surface tension of topramezone solution.

Treatment	Surface tension ^a^
	mN/m
Topramezone with MSO*	33.4±1.1 b
Topramezone alone	65.5±1.4 a
Water	67.2±0.4 a

* MSO is methylated seed oil; ^a^ Values (means ± SE) followed by same letters are not significantly (P≤0.05) different.

**Table 3:** Effect of MSO on topramezone droplet contact angle on the leaf surfaces of giant foxtail and velvetleaf.

Weed	Treatment	Contact angle ^a^
		^0^
Giant foxtail	Topramezone with MSO*	101.1±3.0 b
	Topramezone alone	124.9±3.1 a
	Water	124.8±4.0 a
Velvetleaf	Topramezone with MSO	48.6±4.2 b
	Topramezone alone	66.5±3.0 a
	Water	65.4±4.6 a

* MSO is methylated seed oil; ^a^ Values (means ± SE) followed by same letters are not significantly (P≤0.05) different.

Surface tension and contact angle are two important factors in adjuvant research [37]. Surface tension is determined only by the physical-chemical property of an adjuvant, but contact angle is the result of interaction between droplet and target surface. For adjuvant at normal application rates, the lower the surface tension is, the lower the contact angle is on target surface [37]. A leaf was considered “wettable” if the water contact angle was less than 90 ^0^ and “non-wettable” if the water contact angle was greater than 90 ^0^ [38], [39]. According to this rule, velvetleaf could be classified as “wettable” while giant foxtail as “non-wettable”. Our results showed that MSO could decrease the contact angle on these two kinds of leaves, but decreasing degree was larger on the “wettable” leaf surface (velvetleaf) than on the “non-wettable” leaf surface (giant foxtail).

### Effect of MSO on Spread and Evaporation of Topramezone Droplet on Leaf Surfaces

The droplet of topramezone alone solution almost did not spread on the surface of giant foxtail and velvetleaf leaves till complete evaporation. However, the spreading area increased significantly on the leaves of these two weeds when the solution was mixed with MSO at the concentration 0.3% v/v. After topramezone solution was mixed with MSO, the evaporation time of the droplets shortened significantly on giant foxtail, but did not on velvetleaf. The indexλof treatment topramezone with MSO was significantly higher than those of treatments topramezone alone and water control on both weed species (Table 4).

**Table 4:** Effect of MSO on spread and evaporation of topramezone on leaf surface of giant foxtail and velvetleaf.

Weed	Treatment	Spread area^ a^	Evaporation time^ a^	λ^a^
		cm^2^	Min	min×cm^2^
Giant foxtail	Topramezone with MSO*	0.27±0.03 a	14.66±1.90 b	3.65±0.34 a
	Topramezone alone	0.02±0.00 b	32.42±1.97 a	0.62±0.08 b
	Water	0.01±0.00 b	30.47±2.51 a	0.36±0.05 b
Velvetleaf	Topramezone with MSO	0.08±0.01 a	17.48±0.84 a	1.41±0.20 a
	Topramezone alone	0.05±0.01 b	19.63±1.16 a	0.94±0.12 b
	Water	0.03±0.01 b	17.77±0.61 a	0.55±0.12 b

* MSO is methylated seed oil; ^a^ Values (means ± SE) in the same column followed by same letters are not significantly (P≤0.05) different. λ means the product of the spread area and the evaporation time.

After adjuvant MSO was mixed, increasing spread areas of the droplets is due to the decrease of their contact angles on weed leaves. Larger spread area on giant foxtail than on velvetleaf is probably due to the waxier giant foxtail leaf surface than that of velvetleaf. Sanyal et al. reported that the wax content of velvetleaf was 7.4 µg cm^−2^ and that of green foxtail was 19.1 µg cm^−2^ [40], [41]. In general, a larger droplet spread area and extending liquid state increases pesticide uptake in the target plants. However, these two desirable conditions are contradictory because a larger wetted area always occurred with less evaporation time. Xu et al. introduced the integrated indexλto evaluate droplet spread and resistance to drying [7], [8]. This index is more reasonable to characterize the ability of an adjuvant in increasing spread and decreasing evaporation. In our study, we found the same phenomena of larger spread area but less evaporation time after mixing MSO. However, the indexλvalue of treatment topramezone with MSO was significantly higher than those of treatments topramezone alone and water control on both velvetleaf and giant foxtail. The result was similar to the results of Xu et al., in which MSO was good at increasing indexλof distilled water on both waxy and hairy leaf surfaces [7], [8].

### Effect of MSO on Deposition of Topramezone on Leaf Surfaces

**Figure 2:**
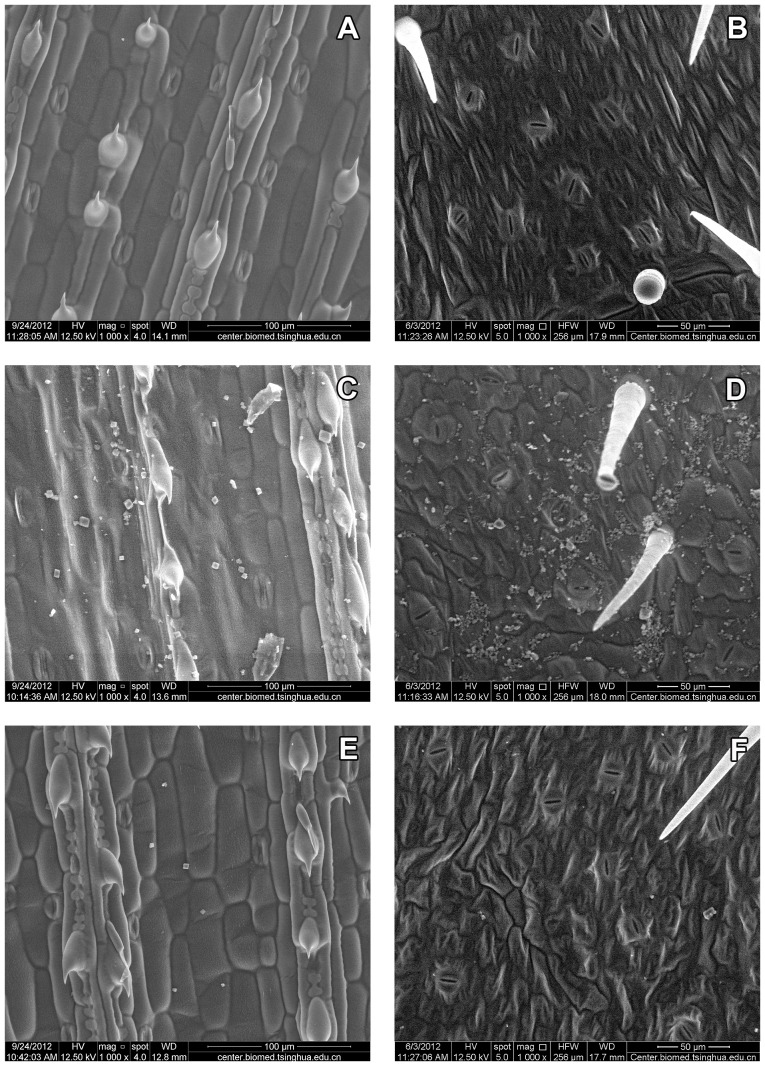
Active ingredient depositions of topramezone on the adaxial sides of giant foxtail and velvetleaf leaves. A, C and E are giant foxtail leaves, and B, D and F are velvetleaf leaves. The upper two (A and B) means blank control, the middle (C and D) means treated with topramezone alone and the lower two (E and F) means treated with topramezone mixed with 0.3% MSO (v/v); topramezone dose is 25.2 g a.i. ha^−1^; control means treated with deionised water; pictures were photographed at 1000× magnification using an environment scanning electron microscope at low vacuum mode at 12.5 kV.

The leaf surface of giant foxtail was relatively more flat and waxy while that of velvetleaf was more hairy (Fig 2). A lot of crystals of active topramezone ingredient were observed on the leaf surface of giant foxtail or velvetleaf treated with topramezone alone. However, only a few crystals were observed on the leaf surfaces of giant foxtail and velvetleaf treated by topramezone mixed with MSO. The results indicated that MSO decreased topramezone crystallization on both weed leaf surfaces.

Due to nearly no spread of droplets of topramezone applied alone, less active ingredient penetrated into the leaf. A lot of active ingredient was deposited and then crystalized on the leaf surface after water evaporation. While in the treatment of topramezone mixed with MSO, the droplet spread significantly larger compared with the treatment of topramezone applied alone, so less active ingredient was crystalized as solid state.

### Effect of MSO on Absorption of Topramezone by Leaves

**Figure 3:**
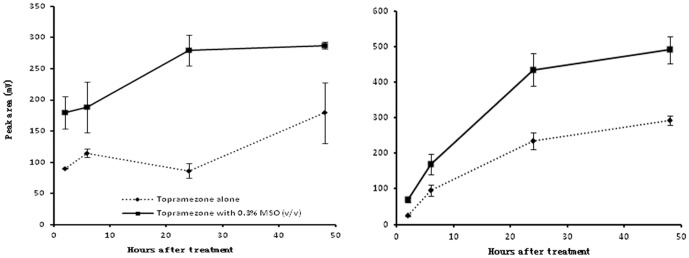
Topramezone absorption of giant foxtail and velvetleaf after treatment. The left is the result of giant foxtail and the right is the result of velvetleaf. Topramezone dose is 100.8^−1^.

Topramezone absorption by giant foxtail and velvetleaf leaves increased from 2 HAT to 48 HAT whether the herbicide was applied alone or with MSO. However, compared with the treatment of topramezone applied alone, the absorption amount was larger when it was mixed with MSO at any sampling times after herbicide application for both weed species. Especially at 24 HAT, the absorption amount of topramezone increased by 68.9% for giant foxtail and by 45.9% for velvetleaf (Fig 3). The result indicated that MSO increased topramezone absorption by both weed leaves.

Grossmann and Ehrhardt found that the adjuvant Dash HC (a kind of MSO) increased the foliar uptake of giant foxtail, sorghum (*Sorghum bicolor*), black nightshade (*Solanum nigrum*) and corn [17], which was similar to our result. Less herbicide is absorbed by the leaf if the herbicide is in a solid rather than in a liquid form [42]. MSO reduced the crystallization of active topramezone ingredient, and then resulted in increasing absorption when toramezone was mixed with MSO.

### Effect of MSO on Translocation of Topramezone in the Plants

**Figure 4:**
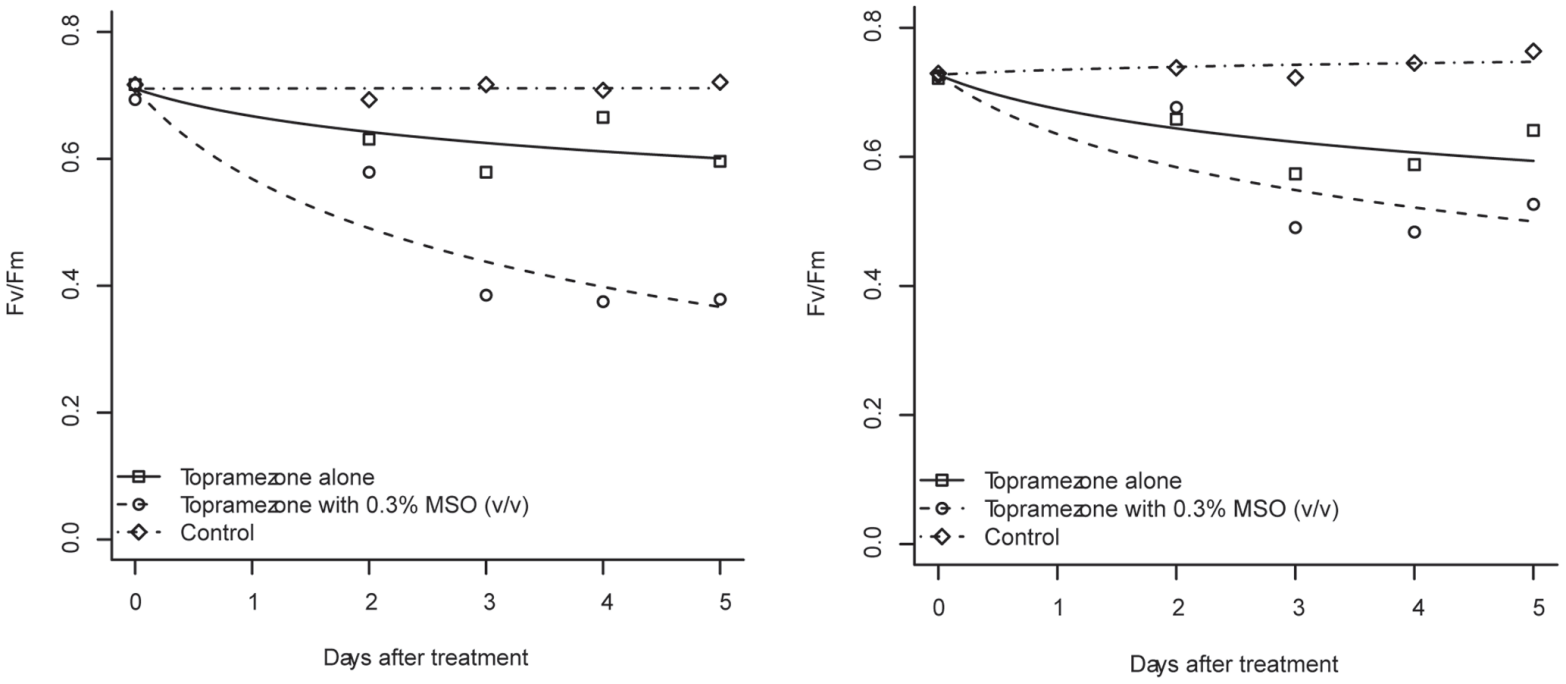
PS II photochemistry maximum quantum efficiency (Fv/Fm) of the leaf of giant foxtail and velvetleaf at different time after herbicide application. The left is the result of giant foxtail and the right is the result of velvetleaf. Topramezone dose is 25.2^−1^; control means treated with tap water.

**Figure 5:**
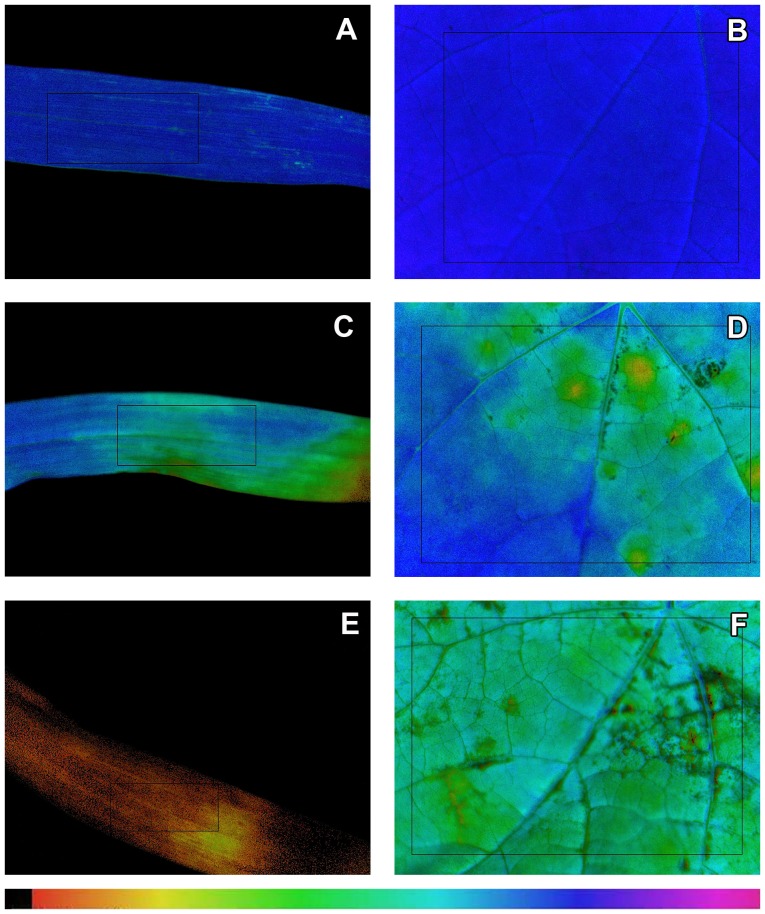
Chlorophyll fluorescence images of leaves of giant foxtail and velvetleaf treated by different solutions 5 days after treatment. A, C and E are giant foxtail leaves, and B, D and F are velvetleaf leaves; the upper two (A and B) means blank control, the middle (C and D) means treated with topramezone alone and the lower two (E and F) means treated with topramezone mixed with 0.3% MSO (v/v); Topramezone dose is 25.2 g a.i. ha^−1^; control means treated with tap water. Maximum quantum efficiency is reduced more when the color turns from blue to red (black).

Lack-of-fit tests showed that equation 3 well described the change of the Fv/Fm value as time (P>0.05). The Fv/Fm values of the coated leaves declined exponentially with time either the plants treated by topramezone alone or by topramezone mixed with MSO. However, the declining speed of the treatment of topramezone with MSO was faster than that of the treatment of topramezone alone (Fig 4). In the case of giant foxtail, 95%-Cl of the curve slope for topramezone with MSO was −0.1532 to −0.0974, while that of topramezone alone was −0.0649 to −0.0091, which meant that they were statistically different with each other (the 95%-CIs did not overlap with each other). In the case of velvetleaf, the slope of the treatment of topramezone with MSO was steeper than that of the treatment of topramezone alone, but their slope 95%-CIs slightly overlapped. The former was −0.1077 to −0.0497, and the latter was −0.0738 to 0.0159. The chlorophyll fluorescence image of the giant foxtail leaf treated by topramezone with MSO was yellow to black while that of the leaf treated by topramezone alone was only light green; the image of the velvetleaf leaf treated by topramezone with MSO was green while that of the leaf treated by topramezone alone was only light green (Fig 5). These results indicated that the active ingredient of topramezone was translocated more and faster from treated leaves to untreated leaves (coated leaves) when this herbicide was mixed with MSO than applied alone, which resulted in more severe and faster photosynthesis inhibition of the untreated leaves.

There is a strong correlation between the translocation and the absorption of a herbicide in the plants [14], [16], [43]. In this study, MSO significantly increased absorption and translocation of topramezone for giant foxtail, but it significantly increased only absorption of this herbicide but not translocation for velvetleaf. This phenomenon needs further study in the future.

For agriculture, the enhancement of herbicide biological efficacy by the addition of adjuvant can contribute to reducing herbicide application rates [44]. The performance of MSO adjuvants is dependent upon the source and composition of the oil, the nature of the herbicide and treated plant species. It has been demonstrated clearly that appropriate combinations of herbicides with adjuvants can greatly enhance the rate and efficiency of herbicides delivery to target sites, and thus the ultimate activity [2], [6]. Our results showed that the combination of methyleated soybean oil adjuvant and topramezone enhanced the herbicide being delivered to the target sites and then provided a really good efficacy on weeds. This kind MSO is also used as an adjuvant for some other herbicides on weed control. In addition, this adjuvant is minimum health risk to humans and the environment because it comes from soybean and has good biodegradation ability.
